# Mechanical Strength of 17 134 Model Proteins and Cysteine Slipknots

**DOI:** 10.1371/journal.pcbi.1000547

**Published:** 2009-10-30

**Authors:** Mateusz Sikora, Joanna I. Sułkowska, Marek Cieplak

**Affiliations:** 1 Institute of Physics, Polish Academy of Sciences, Warsaw, Poland; 2 Center for Theoretical Biological Physics, University of California, San Diego, California, USA; Rensselaer Polytechnic Institute, United States of America

## Abstract

A new theoretical survey of proteins' resistance to constant speed stretching is performed for a set of 17 134 proteins as described by a structure-based model. The proteins selected have no gaps in their structure determination and consist of no more than 250 amino acids. Our previous studies have dealt with 7510 proteins of no more than 150 amino acids. The proteins are ranked according to the strength of the resistance. Most of the predicted top-strength proteins have not yet been studied experimentally. Architectures and folds which are likely to yield large forces are identified. New types of potent force clamps are discovered. They involve disulphide bridges and, in particular, cysteine slipknots. An effective energy parameter of the model is estimated by comparing the theoretical data on characteristic forces to the corresponding experimental values combined with an extrapolation of the theoretical data to the experimental pulling speeds. These studies provide guidance for future experiments on single molecule manipulation and should lead to selection of proteins for applications. A new class of proteins, involving cystein slipknots, is identified as one that is expected to lead to the strongest force clamps known. This class is characterized through molecular dynamics simulations.

## Author Summary

The advances in nanotechnology have allowed for manipulation of single biomolecules and determination of their elastic properties. Titin was among the first proteins studied in this way. Its unravelling by stretching requires a 204 pN force. The resistance to stretching comes mostly from a localized region known as a force clamp. In titin, the force clamp is simple as it is formed by two parallel *β*-strands that are sheared on pulling. Studies of a set of under a hundred proteins accomplished in the last decade have revealed a variety of the force clamps that lead to forces ranging from under 20 pN to about 500 pN. This set comprises only a tiny fraction of proteins known. Thus one needs guidance as to what proteins should be considered for specific mechanical properties. Such a guidance is provided here through simulations within simplified coarse-grained models on 17 134 proteins that are stretched at constant speed. We correlate their unravelling forces with two structure classification schemes. We identify proteins with large resistance to unravelling and characterize their force clamps. Quite a few top strength proteins owe their sturdiness to a new type of the force clamp: the cystein slipknot in which the force peak is due to dragging of a piece of the backbone through a closed ring formed by two other pieces of the backbone and two connecting disulphide bonds.

## Introduction

Atomic force microscopy, optical tweezers, and other tools of nanotechnology have enabled induction and monitoring of large conformational changes in biomolecules. Such studies are performed to assess structure of the biomolecules, their elastic properties, and ability to act as nanomachines in a cell. Stretching studies of proteins [1] are of a particular current interest and they have been performed for under a hundred of systems. Interpretation of some of these experiments has been helped by all-atom simulations, such as reported in refs. [2],[3]. They are limited by of order 100 ns time scales and thus require using unrealistically large constant pulling speeds. However, they often elucidate the nature of the force clamp – the region responsible for the largest force of resistance to pulling, 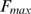. All of the experimental and all-atom simulational studies address merely a tiny fraction of proteins that are stored in the Protein Data Bank (PDB) [4]. Thus it appears worthwhile to consider a large set of proteins and determine their 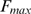 within an approximate model that allows for fast and yet reasonably accurate calculations. Structure-based models of proteins, as pioneered by Go and his collaborators [5] and used in several implementations [6]–[13], seem to be suited to this task especially well since they are defined in terms of the native structures away from which stretching is imposed.

There are many ways, all phenomenological, to construct a structure-based model of a protein. 504 of possible variants are enumerated and 62 are studied in details in ref. [14]. The variants differ by the choice of effective potentials, nature of the local backbone stiffness, energy-related parameters, and of the coarse-grained degrees of freedom. The most crucial choice relates to making a decision about which interactions between amino acids count as native contacts. Comparing 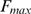 to the corresponding experimental values in 36 available cases selects several optimal models [14]. Among them, there is one which is very simple and which describes a protein in terms of its 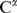 atoms, as labeled by the sequential index 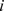. This model is denoted by 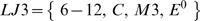 which stands for, respectively, the Lennard-Jones native contact potentials, local backbone stiffness represented by harmonic terms that favor the native values of local chiralities, the contact map in which there are no 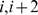 contacts, and the amplitude of the Lennard-Jones potential, 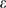, is uniform. The contact map is determined by assigning the van der Waals spheres to the heavy atoms (enlarged by a factor to account for attraction) and by checking whether spheres belonging to different amino acids overlap in the native state [15],[16]. If they do, a contact is declared as native. Non-native contacts are considered repulsive. Application of this criterion frequently selects the 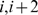 contacts as native. If the contact map includes these contacts the resulting model will be denoted here as 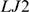. On average, it performs worse than 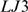 because the 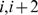 contacts usually correspond to the weak van der Waals couplings as can be demonstrated in a sample of proteins by using a software [17] which analyses atomic configurations from the chemical perspective on molecular bonds. Thus the 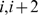 couplings should better be removed from the contact map (in most cases).

The survey to determine 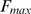 in 7510 model proteins with the number of amino acids, 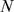, not exceeding 150 and 239 longer proteins (with 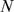 up to 851) has been accomplished twice. First within the 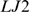 model [18] and soon afterwords within the 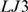 model [19]. The first survey also comes with many details of the methodology whereas the second just presents the outcomes. The two surveys are compared in more details in refs. [14],[20]. The results differ, particularly when it comes to ranking of the proteins according to the value of 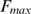, but they mutually provide the error bars on the findings. They both agree, however, on predicting that there are many proteins whose strength should be considerably larger than the frequently studied benchmark – the sarcomere protein titin (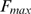 of order 204 pN [21],[22]). Near the top of the list, there is the scaffoldin protein c7A (the PDB code 1aoh) which has been recently measured to have 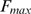 of about 480 pN [23]. Other findings include establishing correlations with the CATH hierarchical classification scheme [24],[25], such as that there are no strong 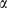 proteins, and identification of several types of the force clamps. The large forces most commonly originate in parallel 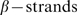 that are sheared [26]. However, there are also clamps with antiparallel 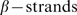, unstructured strands, and other kinds.

The two surveys have been based on the structure download made on July 26, 2005 when the PDB comprised 29 385 entries. Many of them correspond to nucleic acids, complexes with nucleic acids and with other proteins, carbohydrates, or come with incomplete files and hence the much smaller number of proteins that could be used in the molecular dynamics studies. Here, we present results of still another survey which is based on a download of December 18, 2008 which contains 54 807 structure files and leads to 17 134 acceptable structures with 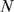 not exceeding 250 (instead of 150). These structures are then analyzed through simulations based on the 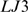 model. The numerical code has been improved to allow for acceleration of calculations by a factor of 2.

The 190 structures (or 1.1% of all structure considered) with the top values of 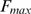 in units of 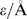 are shown in Table 1 (the first 81 entries for which 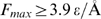) and Table S1 of the SI (proteins ranked 82 through 190), together with the values of titin (1tit) and ubiquitin (1ubq) to provide a scale. As argued in the Materials and Methods section section, the unit of force, 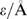, is now estimated to be of order 110 pN. All of the corresponding proteins are predicted to be much stronger than titin and none but two of them (1aho, 1g1k [23]) have been studied experimentally yet. In addition to the types of force clamps identified before, we have discovered two new mechanisms of sturdiness. One of them involves a cysteine slipknot (CSK) and is found to be operational in all of the 13 top strength proteins. In this motif, a slip-loop is pulled out of a cysteine knot-loop. Another involves dragging of a single fragment of the main chain across a cysteine knot-loop. The two mechanisms are similar in spirit since both involve dragging of the backbone. However, in the CSK case, two fragments of the backbone are participating.

**Table 1:** The predicted list of the strongest proteins.

n	PDBid	N	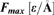	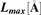	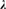	CATH	SCOP
1	**1bmp**	104	**10.2**	23.2	0.01	2.10.90.10	g.17.1.2
2	**1qty**	95	**8.9**	72.1	0.11	2.10.90.10	b.1.1.4
3	**2bhk**	119	**7.3**	26.5	0.67		
4	**1lxi**	104	**7.3**	22.5	0.01		g.17.1.2
5	**1cz8**	107	**6.4**	76.5	0.13	2.10.90.10	b.1.1.1
6	**2gh0**	219	**5.8**	25.9	0.06		
7	**1wq9**	100	**5.5**	72.0	0.10	2.10.90.10	g.17.1.1
8	**1flt**	107	**5.5**	75.6	0.12	2.10.90.10	b.1.1.4
9	**1fzv**	117	**5.4**	90.4	0.12	2.10.90.10	g.17.1.1
10	**2gyz**	100	**5.4**	14.4	0.01		
11	**1rew**	103	**5.3**	21.7	0.01	2.10.90.10	g.7.1.3
12	**1m4u**	139	**5.3**	52.1	0.07	2.10.90.10	g.17.1.2
13	**1vpf**	94	**5.3**	68.1	0.11	2.10.90.10	g.17.1.1
14	**1c4p**	137	**5.1**	106.0	0.12	3.10.20.180	d.15.5.1
15	**1qqr**	138	**5.0**	110.3	0.12	3.10.20.180	d.15.5.1
16	**3bmp**	114	**5.0**	33.0	0.03	2.10.90.10	g.17.1.2
17	**1j8s**	193	**4.9**	77.9	0.03	2.60.40.1370	b.2.3.3
18	**1wq8**	96	**4.9**	82.6	0.11	2.10.90.10	g.17.1.1
19	**1j8r**	193	**4.8**	77.7	0.03	2.60.40.1370	b.2.3.3
20	**1f3y**	165	**4.8**	284.7	0.43	3.90.79.10	d.113.1.1
21	**2vpf**	109	**4.7**	79.3	0.11	2.10.90.10	g.17.1.1
22	**2h64**	105	**4.6**	29.4	0.03		g.7.1.3
23	**1kdm**	177	**4.6**	309.4	0.45	2.60.120.200	b.29.1.4
24	**1q56**	195	**4.5**	473.2	0.62	2.60.120.200	b.29.1.4
25	**1rv6**	94	**4.5**	67.7	0.11	2.10.90.10	b.1.1.4
26	**1waq**	104	**4.5**	20.1	0.01		
27	**1reu**	103	**4.5**	20.4	0.01	2.10.90.10	g.17.1.2
28	**1tgj**	112	**4.4**	45.9	0.07	2.10.90.10	g.17.1.2
29	**2pbt**	133	**4.4**	219.9	0.39		
30	**2h62**	104	**4.4**	24.3	0.02		g.7.1.3
31	**1tgk**	112	**4.4**	44.6	0.07	2.10.90.10	g.17.1.2
32	**2fzl**	197	**4.4**	49.7	0.02		c.37.1.19
33	**1qu0**	181	**4.3**	156.9	0.22	2.60.120.200	b.29.1.4
34	**1f5f**	172	**4.3**	186.2	0.28	2.60.120.200	b.29.1.4
35	**1dzk**	148	**4.3**	110.3	0.16	2.40.128.20	b.60.1.1
36	**1aoh**	147	**4.3**	77.1	0.01	2.60.40.680	b.2.2.2
37	**1vsc**	196	**4.3**	238.3	0.24	2.60.40.10	b.1.1.3
38	**2c7w**	96	**4.2**	184.2	0.45	2.10.90.10	
39	**2gyr**	97	**4.2**	27.1	0.05	2.10.90.10	
40	**1dzj**	148	**4.2**	111.0	0.16	2.40.128.20	b.60.1.1
41	**2sak**	121	**4.2**	76.0	0.10	3.10.20.130	d.15.5.1
42	**2bzm**	129	**4.2**	124.3	0.24		
43	**2pq1**	134	**4.1**	222.6	0.39		
44	**1nwv**	129	**4.1**	129.8	0.13	2.10.70.10	g.18.1.1
45	**1e5g**	120	**4.1**	133.1	0.17	2.10.70.10	g.18.1.1
46	**2ick**	220	**4.1**	462.5	0.54		
47	**1gvl**	223	**4.1**	114.9	0.09	2.40.10.10	b.47.1.2
48	**1tgs**	225	**4.1**	122.3	0.10	2.40.10.10	b.47.1.2
49	**1u20**	196	**4.0**	408.5	0.53		d.113.1.1
50	**1cui**	197	**4.0**	422.8	0.55	3.40.50.1820	c.69.1.30
51	**1ffd**	197	**4.0**	423.0	0.55	3.40.50.1820	c.69.1.30
52	**1kdk**	177	**4.0**	357.2	0.53	2.60.120.200	b.29.1.4
53	**2icj**	219	**4.0**	455.9	0.53		
54	**3dd5**	194	**4.0**	403.3	0.53		
55	**1cug**	197	**4.0**	422.6	0.55	3.40.50.1820	c.69.1.30
56	**1b0o**	161	**4.0**	237.3	0.36	2.40.128.20	b.60.1.1
57	**1xza**	197	**4.0**	422.9	0.55	3.40.50.1820	c.69.1.30
58	**1vcd**	126	**4.0**	199.7	0.37		d.113.1.1
59	**1cuw**	197	**4.0**	422.9	0.55	3.40.50.1820	c.69.1.30
60	**1xzi**	197	**4.0**	422.9	0.55	3.40.50.1820	c.69.1.30
61	**1cus**	197	**4.0**	423.3	0.55	3.40.50.1820	c.69.1.30
62	**1cuf**	197	**4.0**	423.1	0.55	3.40.50.1820	c.69.1.30
63	**2a7h**	223	**4.0**	114.7	0.10	2.40.10.10	b.47.1.2
64	**1cq3**	224	**4.0**	128.0	0.12	2.60.240.10	b.27.1.1
65	**1ffc**	197	**3.9**	421.6	0.55	3.40.50.1820	c.69.1.30
66	**1vc9**	126	**3.9**	199.1	0.37		d.113.1.1
67	**1cua**	197	**3.9**	423.0	0.55	3.40.50.1820	c.69.1.30
68	**1xzl**	197	**3.9**	423.1	0.55	3.40.50.1820	c.69.1.30
69	**2faw**	250	**3.9**	250.8	0.25		
70	**2vn5**	142	**3.9**	49.2	0.02		
71	**1cux**	197	**3.9**	421.5	0.55	3.40.50.1820	c.69.1.30
72	**1cuh**	197	**3.9**	421.6	0.55	3.40.50.1820	c.69.1.30
73	**2dsd**	195	**3.9**	429.7	0.56		
74	**2f3c**	221	**3.9**	113.5	0.10	2.40.10.10	b.47.1.2
75	**1xzj**	197	**3.9**	421.8	0.55	3.40.50.1820	c.69.1.30
76	**1xzf**	197	**3.9**	421.0	0.55	3.40.50.1820	c.69.1.30
77	**2g7i**	124	**3.9**	106.6	0.10		
78	**1g1k**	143	**3.9**	52.0	0.02	2.60.40.680	b.2.2.2
79	**1cuc**	197	**3.9**	421.3	0.55	3.40.50.1820	c.69.1.30
80	**1xzk**	197	**3.9**	422.5	0.55	3.40.50.1820	c.69.1.30
81	**1i04**	159	**3.9**	231.7	0.34	2.40.128.20	b.60.1.1
3144	**1ubq**	76	**2.2**	47.9	0.04	3.10.20.90	d.15.1.1
3580	**1tit**	89	**2.1**	55.3	0.04	2.60.40.10	b.1.1.4

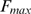
 is obtained within the 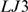 model at the pulling velocity of 0.005 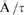. The first column indicates the ranking of a model protein, the second – the PDB code, and the third – the number of the amino acids that are present in the structure used. 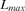 denotes the end-to-end distance at which the maximum force arises. 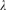 is the corresponding dimensionless location defined as 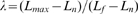, where 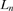 is the native end-to-end distance and 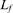 corresponds to full extension. The last two columns give the leading CATH and SCOP codes. The survey is performed based strictly on the PDB-assigned structure codes. It may happen that the structure of a protein has been determined several times and then each of these determinations leads to its own value of 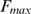. In this case, one may derive the best estimate either by picking the best resolved structure or by making (weighted) averages over all related structures.

We make a more systematic identification of the CATH-classified architectures that are linked to mechanical strength and then analyze correlations of the data to the SCOP-based grouping (version 1.73) [27]–[29]. The previous surveys did not relate to the SCOP scheme.

We identify the CATH-based architectures and SCOP-based folds that are associated with the occurrence of a strong resistance to pulling. A general observation, however, is that each such group of structures may also include examples of proteins that unravel easily. The dynamics of a protein are very sensitive to mechanical details that are largely captured by the contact map and not just by the appearance of a structure. On the other hand, if one were to look for mechanically strong proteins then the architectures and folds identified by us should provide a good starting point. We also study the dependence of 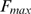 on the pulling velocity and characterize the dependence on 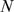 through distributions of the forces.

The current third survey has been performed within the same 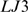 model as the second survey [19]. However, we reuse and extend it here because the editors of Biophysical Journal retracted the second survey [30]. All of the values of 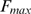 are deposited at the website www.ifpan.edu.pl/BSDB (Biomolecule Stretching Database) and can by accessed by through the PDB structure code.

## Results/Discussion

### Distribution of Forces

**Figure 1:**
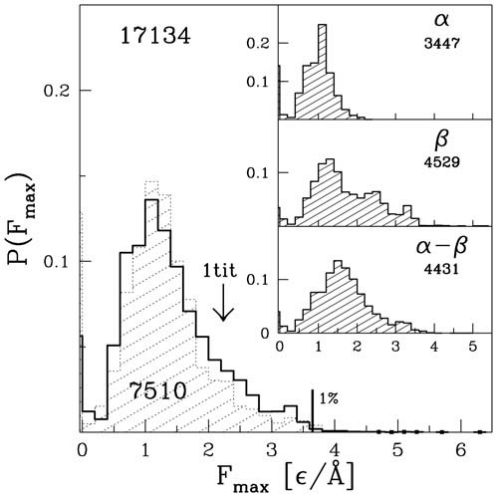
Probability distribution of the maximal forces obtained in the set of 17 134 model proteins (solid line). The shaded histogram corresponds to the 7510 proteins studied in ref. [19]. The insets show similar distributions for the CATH-based classes indicated. The numbers underneath the class symbols give the size of the set of the proteins considered.

The distribution of all values of 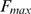 for the full set of proteins is shown in Figure 1. Despite the larger limit on 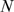 now allowed, the distribution is rather similar to that obtained in ref. [19] for the smaller number of proteins (and with the smaller sizes). The similarity is primarily due to the fact that the size related effects, discussed below, are countered by new types of proteins that are now incorporated into the survey. The distribution is peaked around 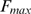 of 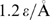 which constitutes about 60% of the strength associated with titin. The distribution is non-Gaussian: it has a zero-force peak and a long force tail. The zero-force peak arises in some proteins with the covalent disulphide bonds. In the model, such bonds are represented by strong harmonic bonds. Stretching of such a protein may not result in any force peak before a disulphide bond gets stretched indefinitely and hence 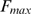 is considered to be vanishing then. The tail, on the other hand, corresponds to the strong proteins. The top strongest 1.1% of all proteins are listed in Tables 1 (in the main text) and S1 (in the SI).

The insets of Figure 1 show similar distributions for proteins belonging to the particular CATH-based classes. There are four such classes: 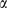, 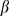, 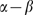 and proteins with no apparent secondary structures. It is seen that none of the 3240 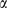 proteins exceeds the peak force obtained for titin within our model. This observation is in agreement with experiments on several 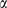 proteins that are listed in the Materials and Methods section. All strong proteins are seen to involve the 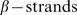. The peak in the probability distribution for the 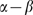 proteins is observed to be shifted towards the bigger values of 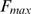 compared to the one for the 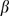 proteins. At the same time, the high force tail of the distribution for the 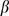 proteins is substantially more populated than the corresponding tail for the 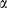 proteins.

**Figure 2:**
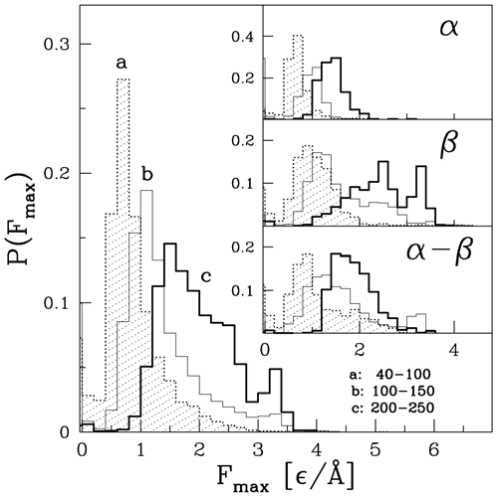
Similar to Figure 1 but for proteins belonging to specific ranges of the sequential sizes, as indicated by the symbols a, b, and c.

Figure 2 is similar to Figure 1 in spirit, but now the structures are split into particular ranges of the protein sizes: 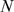 between 40 and 100 (the dotted line), between 100 and 150 (thin solid line), and between 200 and 250 (the thick solid line). The curve for the range from 150 to 200 is in-between the curves corresponding to neighboring ranges and is not shown in order not to crowd the Figure. The distributions are seen to be shifting to the right when increasing the range of the values of 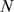 indicating, that the bigger the number of amino acids, the more likely a protein is to have a large value of 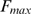. This observation holds for all classes of the proteins, as evidenced by the insets in Figure 2.

In most cases, the major force peak arises at the begining of stretching where the Go-like model should be applicable most adequately. One can characterize the location of 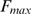 during the stretching process by a dimensionless parameter 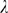 which is defined in terms of the end-to-end distance, as spelled out in the caption of Table 1. This parameter is equal to 0 in the native state and to 1 in the fully extended state. In 25% of the proteins studied in this survey, 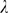 was less than 0.25 and in 52% – les than 0.5. There are very few proteins with 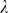 exceeding 0.8.

Table 1 does not include any (non-cysteine-based) knotted proteins. The full list of 17 134 proteins contains 42 such proteins but they come with moderate values of 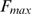. However, knotted proteins with 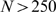 may turn out to have different properties.

### Biological properties of the strongest proteins

A convenient way to learn about the biological properties listed in Tables 1 and S1 is through the Gene Ontology data base [31] which links such properties with the PDB structure codes. The properties are divided into three domains. The first of these is “molecular function” which describes a molecular function of a gene product. The second is “biological processes” and it covers sets of molecular events that have well defined initial and final stages. The third is “cellular component” and it specifies a place where a given gene product is most likely to act.

The results of our findings are summarised in Table 2. It can be seen, that most of the 190 strongest proteins are likely to be found in an extracellular space where conditions are much more reducing than within cells. Larger mechanical stability is advantageous under such conditions. 90 out of the strongest proteins exhibit hydrolase activity. 39 of these 90 are serine-type endopeptidases. These findings seem to be consistent with expectations regarding proteins endowed with high mechanical stability. For instance, proteases, which are well represented in Table 2 should be more stable to prevent self-cleavage.

**Table 2:** Gene Ontology terms for the top 190 proteins.

Domain	GO identifier	Term name	No. of structures	Example
**Molecular function**	GO:0016787	hydrolase activity	90	1f3y
	GO:0003824	catalytic activity	70	1gvl
	GO:0004252	serine-type endopeptidase activity	39	1c4p
	GO:0008083	growth factor activity	25	1bmp
**Biological process**	GO:0006508	proteolytic activity	34	2a7h
	GO:0007586	digestion	32	1bra
**Cellular component**	GO:0005576	extracellular region	122	1vpf, 1aoh
	GO:0005515	protein binding	70	1bmp

### CATH-based architectures

The classification of proteins within the CATH (Class, Architecture, Topology, Homology) data base is done semi-automatically by applying numerical algorithms to structures that are resolved better than within 4 Å [24],[25]. The four classes of proteins in the CATH system are split into architectures, depending on the overall spatial arrangement of the secondary structures, the numbers of 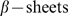 in various motifs, and the like. The next finer step in this hierarchical scheme is into topologies and it involves counting contacts between amino acids which are sequentially separated by more than a treshold. The further divisions into homologous superfamilies and then sequence family levels involve studies of the sequential identity.

**Figure 3:**
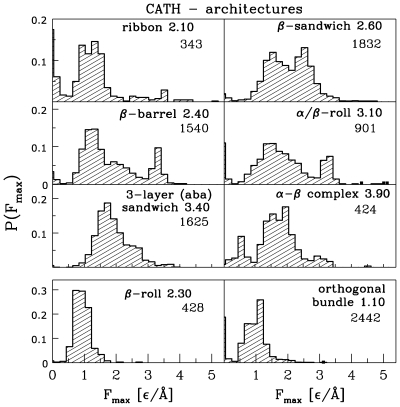
The top six panels show probability distributions of 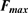 for the architectures that contribute to the pool of proteins with large forces. The architectures are indicated by their names and the accompanying CATH numerical symbol. The numbers underneath the symbols of the architecture inform about the number of cases contributing to the distribution. The bottom two panels show examples of architectures that are predicted to yield only small values of 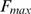.

We have found that only six architectures contribute to 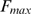 larger than 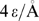. These are ribbons – 2.10 (41.8% of the proteins listed in Table 1), 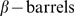 – 2.40 (8.9%), 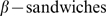 – 2.60 (16.3%), 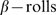 – 3.10 (5.4%), 3-layer (aba) sandwiches – 3.40 (5.4%), and these with no CATH classification to date (21.8%). The corresponding distributions of forces are shown in the top six panels of Figure 3 and the topologies involved are listed and named in Table 3.

**Table 3:** CATH classes (C), architectures (A), and topologies (T) contributing to the top strength proteins.

C	A	T	Strong			All			Root name
2.			57.3%			26.4%			Mainly 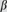
	**2.10**			**17.3%**			2.0%		**Ribbon**
		2.10.70			5.2%			0.1%	Complement Module, domain 1
	**2.40**			**25.7%**			8.9%		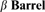
		2.40.10			21.5%			2.9%	Thrombin,subunit H
	**2.60**			**14.2%**			10.6%		**Sandwich**
		2.60.40			3%			7%	Immunoglobulin-like
3.			26.8%			25.8%			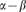
	**3.10**			**8.4%**			5.2%		**Roll**
		3.10.20			2.6%			1.3%	Ubiquitin-like (UB roll)
		3.10.130			5.7%			1.0%	P-30 Protein
	**3.40**			**17.9%**			9.4%		**3-Layer (aba) Sandwich**
		3.40.50			17.9%			5.6%	Rossmann fold
X			15.7%			26.6%			

The percentages indicated in the column denode by “Strong” are relative the top 190 proteins listed in Table 1. X corresponds to proteins not listed in CATH.

Examples of architectures that are dominant contributors to a low force behavior are the 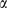 orthogonal bundle (the right bottom panel of Figure 3), the 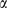 up-down bundle, and the 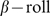 (the left bottom panel of Figure 3).

### SCOP-based classes and folds

The SCOP (Structural Classification of Proteins) data base [27]–[29] is curated manually and it relies on making comparisons to other structures through a visual inspection. This classification scheme is also hierarchical and the broadest division is into seven classes and three quasi-classes. The classes are labelled 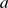 through 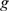 and these are as follows: mainly 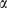 (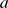), mainly 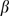 (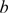), 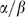 which groups proteins in which helices and 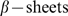 are interlaced (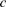), 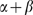 with the helices and 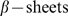 grouped into clusters that are separated spatially (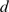), multidomain proteins (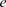), membrane and cell-surface proteins (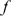), and small proteins that are dominated by disulphide bridges or the heme metal ligands (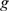). The quasi-classes are labelled 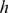 through 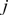 and they comprise coiled-coil proteins (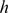), structures with low resolution (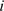), and peptides and short fragments (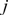). The classes are then partitioned into folds that share spatial arrangement of secondary structures and the nature of their topological interlinking. Folds are then divided into superfamilies (same fold but small sequence identity) and then families (two proteins are said to belong to the same family if their sequence identity is at least 30%). Families are then divided into proteins – a category that groups similar structures that are linked to a similar function. Proteins comprise various protein species.

Each structure assignment comes with an alphanumeric label, as shown in Tables 1, S1, and 4 which reflects the placement in the hierarchy. At the time of our download, there have been 92 972 entries in the SCOP data base that are assigned to 34 495 PDB structures. These entries are divided into 3464 families, 1777 superfamilies and 1086 unique folds. A given structure may have several entry labels but the dominant assignment is listed first. We use the primary assignment in our studies. The same rule is also applied to the CATH-based codes.

**Table 4:** SCOP classes (C) and folds (F) contributing to the top strength proteins.

C	F	Strong		All		Root name	Description
b.		40.5%		22.7%		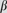	
	**b.47**		**21.5**%		2.7%	SMAD/FHA domain	sandwich; 11 strands in 2 sheets; greek-key
c.		17.9%		9%		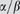	Mainly parallel 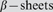 ( 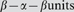 )
	**c.69**		**15.7**%		0.3%	Pyruvate kinase C-terminal domain-like	3 layers: a/b/a; mixed 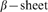 of 5 strands, order 32145, strand 5 is antiparallel to the rest
d.		11.05%		18.9%		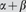	Mainly antiparallel 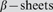 (segregated 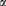 and 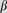 regions)
	**d.5**		**5.8**%		0.9%	RNase A-like	contains long curved 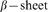 and 3 helices
	**d.113**		**2.6**%		0.2%	DsrC, the 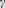 subunit of dissimilatory sulfite reductase	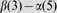 ; meander 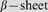 packed against array of helices
g.		13.7%		4.9%		Small proteins	Usually dominated by metal ligand, heme, and/or disulfide bridges
	**g.17**		**5.2**%		0.1%	Necrosis inducing protein 1, NIP1	disulfide-rich fold; 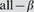 ; duplication: contains two structural repeats
	**g.18**		**6.3**%		0.2%	Trefoil/Plexin domain-like	disulfide-rich fold; common core is 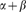 with two conserved disulfides
X		16.3%		27.4%			

X corresponds to proteins not listed in SCOP.

**Figure 4:**
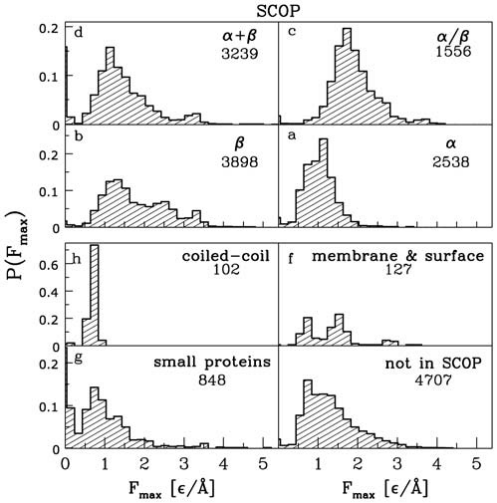
Distributions of 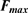 for the SCOP-based classes for which there are more than 60 structures that could be used in molecular dynamics studies. The cases that are not shown are: class e (27 structures), quasi-class i (5 structures), and quasi-class j (52 structures). The bottom right panel corresponds to structures which have no assigned SCOP-based structure label. The numbers indicate the corresponding numbers of structures studied.

Figure 4 shows the distributions of forces for the SCOP-based classes of proteins. The results are consistent with the CATH-based classes since the 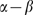 class of CATH basically encompasses the 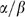 and 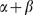 classes of SCOP. However, there are proteins which are classified only according to one of the two schemes. Thus there are 4431 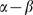 proteins out of which only the total of 3368 is SCOP-classified as belonging to the 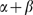 and 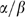 classes. At the same time, the total of the proteins in the 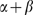 and 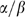 classes we have is 4795.

It should be noted that the peak in the distribution for 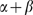 is shifted to higher forces by about 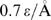 from the peak for 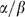. At the same time, the zero-force peak is virtually absent in 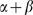. The SCOP-based classification also reveals that its class 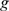 contributes across the full range of forces and, in particular, it may lead to large values of 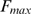. It should be noted, as also evidenced by Table 1, that there is a substantial number of strong proteins that has no class assignment.

**Figure 5:**
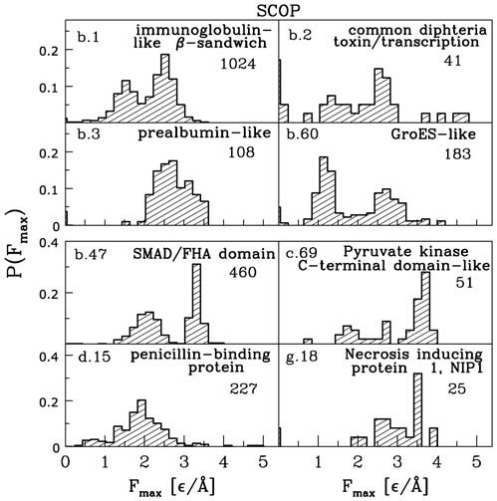
Distributions of 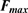 for eight folds that may give rise to a large resistance to pulling.

**Figure 6:**
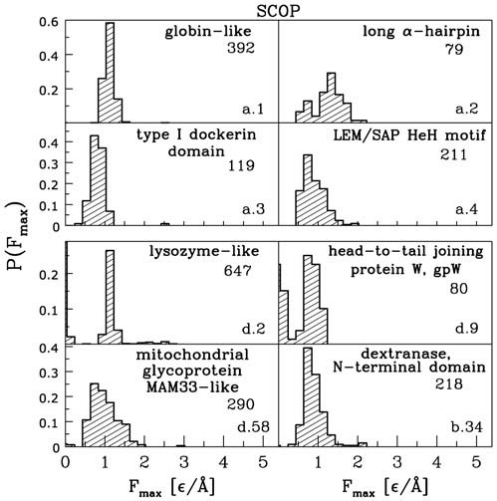
Distribution of 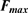 for eight folds that are likely to yield a small resistance to pulling.

Figures 5 and 6 refer to the distributions of 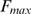 across specific folds. The first of these presents results for the folds that give rise to the largest forces. The names of such folds are specified in Figure 5. The percentage-wise assessment of the folds contributing to big forces is presented in Table 4. The top contributor is found to be the b.47 fold (SMAD/FHA domain). Figure 6 gives examples of folds that typically yield low forces.

It is interesting to note that distributions corresponding to some folds are distinctively bimodal, as in the case of the SMAD/FHA fold (b.47). This particular fold is dominated by SMAD3 MH2 domain (b.47.1.2; 352 structures) which contributes both to the high and low force peaks in the distribution. The remaining domains (b.47.1.1, b47.1.3, and b47.1.4) contribute only to the low force peak. The dynamical bimodality of the b.47.1.2 fold can be ascribed to the fact that the strong subset comes with one extra disulphide bond relative to the weak subset. This extra bond provides substantial additional mechanical stability when stretching is accomplished by the termini. We illustrate sources of this bimodality in the SI (Figure S1) for two proteins from this fold: 1bra which is strong and 1elc which is weak. In ref. [18], we have noted that various sets of proteins with identical CATH codes (e.g., 3.10.10) may give rise to bimodal distributions without any dynamical involvement of the disulphide bonds. The reason for this is that even though the contact maps for the two modes are similar, the weaker subset misses certain longer ranged contacts which pin the structure. Mechanical stability is more sensitive to structural and dynamical details than are not provided by standard structural descriptors.

### Force clamps

#### Shearing motif

**Figure 7:**
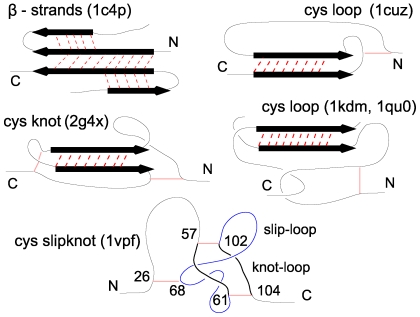
Examples of force clamps found in the top strength proteins. The relevant disulphide bonds are shown in gray shade. The PDB codes of the examples of the proteins that show the particular type of a clamp are indicated. In the case of the CSK, the numbers indicate sequential locations of the amino acids participating in a disulphide bridge in the 13-ranked 1vpf.

The most common type of the force clamp identified in the literature is illustrated in the top left panel of Figure 7 corresponding to the 14th-ranked protein 1c4p. In this case, the strong resistance to pulling is due to a simultaneous shearing of two 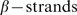 which are additionally immobilised by short 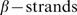 that adhere to the two strands. Similar motifs appears in 1qqr(15), 1j8s(17), 1j8r(19), 1f3y(20), 2pbt(29), 2fzl(15), 1aoh(19), where the number in brackets indicate ranking as shown in Table 1. It is interesting to note that the 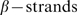 responsible for the mechanical clamp in 1j8s and 1j8r display an additional twist. Undoing the twist enhances 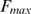. (There is a similar mechanism that seems to be operational in the case of a horseshoe conformation found in ankyrin [32],[33]). The force clamps are identified by investigating the effect of removal of various groups of contacts on the value of 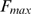 [12],[18].

There are, however, new types of the force clamps that we observe in the proteins listed in Tables 1 and S1. They arise from entanglements resulting from the presence of the disulphide bonds which cannot be ruptured by forces accessible in the atomic force microscopy. We note that about 2/3 of the proteins listed in Tables 1 and S1 contain the disulphide bonds. Many of these bonds do not carry much of dynamical relevance when pulling by the termini. However, in certain situations they are the essence of the force clamp. The disulphide bonds have been already identified as leading to formation of the cystein knot (CK) motifs [34],[35] (such proteins are found in the toxins of spiders and scorpions) and the cyclic CK motifs [36],[37]. Here, we find still another motif – that of the CSK which is similar to that found in slipknotted proteins [38]–[40] which do not conatin the disulphide bonds. This motif is found in the top 13 proteins. The cysteine loop, knot, and slipknot motifs are shown schematically in the remaining panels of Figure 7. It is convenient to divide these motifs into two categories: shallow (S) and deep (D) (according to the classification used for knotted proteins [41],[42]), depending on whether the motif is spanning most of the sequence or is instead localized in its small fraction.

#### Shearing connected with a cysteine loop

**Figure 8:**
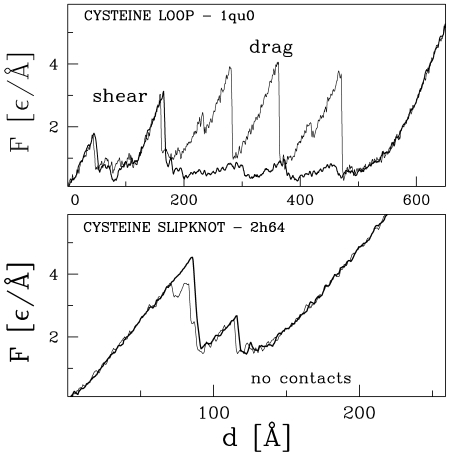
Examples of the force patterns corresponding to proteins with the disulphide bonds.

In this case, the mechanical clamp arises from shearing between a 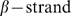 belonging to a deep cysteine loop and another strand located outside the loop (the left bottom panel of Figure 7). Existence of the disulphide bond before the shearing motif allows to decompose direct tension onto the 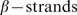 making the protein resist stretching much more effectively than what would be expected from a simple shearing motif. Additionally, the disulphide bonds prevent an onset of any rotation in the protein conformation which otherwise might form an opportunity for unzipping. This motif appears in 1dzj(40,D) 1vsc(37,D), 1dzk(35,D), 1i04 (81,D), 1hqp(83,D), 1oxm(98,D), 2a2g (175,D), 2boc(179,D), and many other proteins. The middle panel of Figure 8 gives an example of the corresponding force (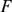) – displacement (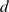) pattern as obtained for 1dzj.

#### Shearing and dragging out of a cysteine loop

This motif consists of two parts. The first is formed by a rather small and deep cysteine loop which is located very close to one terminus with the second terminus located across the cysteine loop. The motif arises when almost all of the protein backbone is dragged across the cysteine loop on stretching. A protein structure also contains a few 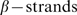 which get sheared before dragging takes place. This motif is seen in 1kdm(23,D), 1q56(24,D), 1qu0(33,D), 1f5f(34,D) and this geometry of pulling we call geometry I. It should be pointed out that, in all such cases, pulling by the N terminus takes place within (or very near) the plane formed by the cysteine loop. A small change in such a geometry, e.g. the one arising from pulling not by the last amino acid but by the penultimate bead, may cause getting out of the cystein loop and result in a very different unfolding pathway with a distinctly different value of 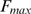. In this other kind of pulling set up, denoted as geometry II, the loop is bypassed and the resistance to pulling is provided only by the shearing mechanism.

**Figure 9:**
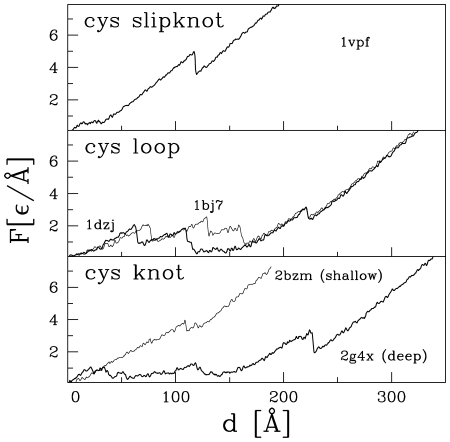
Top: Two trajectories arising in protein 1qu0. Dragging occurs when the backbone is pulled across the cysteine loop. Shearing occurs when the pull across the cystein loop does not take place. Bottom: The force-displacement pattern corresponding to the CSK force clamp in 2h64 (thick line). The thin line shows the corresponding pattern when one removes the attractive contacts that are slipknot related.

Dragging arises from overcoming steric constraints and generates an additional contribution to the strength of the standard shearing mechanical clamp. By using geometry II and also by eliminating the native contacts between the sheared 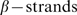 we can estimate the topological contribution of the dragging effect on the value of 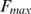. For proteins 1kdm, 1q56, 1qu0, 1f5f, it comes out to be around 25%. The force 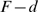 patterns corresponding to these two geometries of pulling are shown in top panel of Figure 9.

In the survey, there are other proteins which also have disulphide bonds and belong to the 2.60.120.200 category. These proteins have a cysteine which is either very shallow or deep, but is located in the middle of the protein backbone so that there is no possibility to form a long 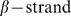. In this case, the dragging effects are much smaller. For instance, for 1pz7(D) and 1cpm(S), 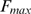 is close to 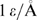.

#### Shearing inside of a cysteine knot

This motif is created by a loosely packed CK (two or more spliced cysteine loops) with at least two parallel 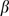 strands that are present within the knot. Pulling protein by termini exerts tension on the entire CK and thus produces an indirect shearing force on the 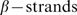 inside the entangled part of the protein. In this case, elimination of the native contacts between the 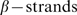 reduces 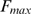 only partially indicating that the mechanical clamp is created also by the CK. A simple CK is also found in 2bzm(42) and many other proteins, e.g. in 2g7i(77,S), 1hfh103,S), 2g4x(136,D), 2g4w(169,D). The 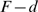 patterns for 2bzm and 2g4x are shown in the bottom panel of Figure 8. More complex structures or higher order CKs (with more than two cystein bonds) can be identified in 1afk(85), 1afl(117), or 1aqp(135). Inside this group of proteins there are also examples of proteins – 1qoz(88,S) – in which a cysteine loop is braided to a CK by some native contacts.

#### Cysteine slipknot force-clamp is observed in the strongest 13 proteins

The top strength protein is 1bmp (bone morphogenic protein) with the predicted 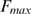 of 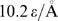, which should correspond to about 1100 pN (see Materials and Methods). This strength should be accessible to standard experiments as the atomic force microscopy has been already used to rupture covalent N-C and C-C bonds by forces of 1500 and 4500 pN respectively [43].

In our discussion, we focus on the 13-ranked 1vpf (a vascular endothelial growth factor) with the predicted 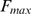 of 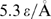. The CSK motif arises from two loops [40]: the knot-loop and the slip-loop, where the slip-loop can be threaded across the knot-loop. One needs at least three disulphide bonds for this motif to arise.

In the case of the 1vpf, the knot-loop is created by the disulphide bonds between amino acids 57 and 102, 61 and 104, and the protein backbone between amino acids 57–61 (GLY,GLY,CYS) and 102–104 (GLU). The slip-loop is created by the protein backbone between sites 61–102 and is stabilized by 12 hydrogen bonds between two parallel 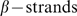. In the CSK motif, the force peak is due to dragging of a slip- loop through the knot-loop making the native hydrogen contacts only marginally responsible for the mechanical resistance. Thus the force peak arises, to a large extent, from overcoming steric constraints, i.e. it is due to repulsion resulting from the excluded volume. The 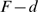 pattern for this novel type of a force clamp is shown in the top panel of Figure 8. Another example of such a pattern for a CSK is shown in the bottom panel of Figure 9 for the 22nd ranked 2h64 (a human transforming growth factor). The leading role of the steric constraints is verified by checking the reduction of the 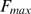 when all the slipknot-related contacts (inside the slip-loop and between the slip-loop and the knot-loop) are converted to be purely repulsive. As a result of this bond removal, the force peak persists, though it gets shifted and becomes smaller. This is summarized in Table S2 in the SI. It is a new and unexpected result.

Another way to establish the role of the CSK motif is to create the disulphide-deficient mutants, as accomplished experimentally [44] for 1vpf. The two mutants, 1mkk (C61A and C104A) and 1mkg (C57A and C102A), have structures similar to 1vpf but contain no knot-loops and thus there is no slipknot. Muller et al. [44] show that the mutants' thermodynamic stability is not reduced but their folding capacity is. Our work shows that the mutants have a reduced resistance to pulling compared to 1vpf: 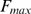 drops from 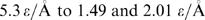 for 1mkk and 1mkg respectively.

We note that the CSK topology is a subgroup inside the CK class (represented mostly by 2.10.90.10) and the CSK force clamp need arise for a particular way of pulling. For instance, proteins 1afk(68), 1afl(100) or 1aqp(118) have up to four disulphide bonds and yet the CSK motif does not play any dynamical role in pulling by the terminal amino acids. In the case of the CSK, we observe a formidable dispersion in the values of 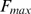. For example, it ranges between 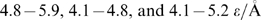 for various trajectories in 1vpf, 2h64, and 2c7w respectively. We now examine the CSK geometry in more details.

#### Cysteine slipknot motif is distinct from the slipknot motif in several ways

**Figure 10:**
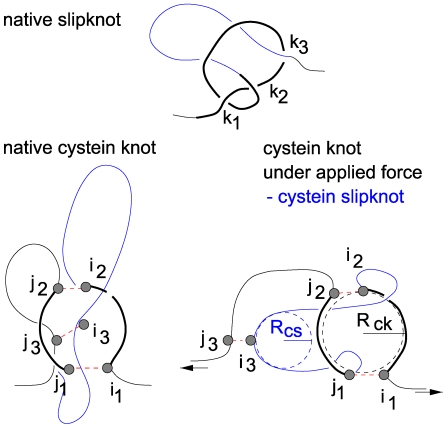
Geometry of a slipknot and a cystein slipknot. The top panel corresponds to a genuine slipknot. The bottom left panel is a schematic representation of the native geometry that yields the cystein slip-knot on stretching. The resulting cystein slipknot motif is shown in the bottom right panel.

The left-most panel of Figure 10 shows a slipknot with three intersections at sequential locations 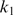, 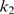, and 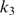. This geometry is topologically trivial since when one pulls by the termini, the apparent entanglement may untie and become a simple line. The entanglement would form the trefoil knot if the 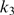 intersection was removed by redirecting the corresponding segment of the chain (thin line) away from the 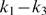 loop. Such slipknot motifs have been observed in native states of several proteins [38]–[40]. In contrast, the CSKs are not present in the native state but arise as a result of mechanical manipulation. The middle panel of Figure 10 shows a schematic representation of a native conformation with three cysteine bonds: between 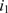 and 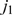, between 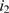 and 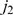, and between 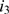 and 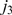. The 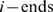 of the bonds are counted as being closer to the N-terminus. The three bonds are in a specific arrangement as shown in the panel. In particular, the 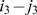 bond must cross the loop 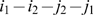. This loop consists of two pieces of the backbone (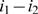 and 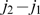) that are linked to form a closed path by the two remaining cysteine bonds – it is the cysteine knot-loop. The average radius of this loop is denoted by 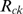.

The arrangement shown in the middle panel has no entanglements that could be considered as knots in the topolgical sense. However, on pulling by the termini, the chain segment adjacent to 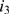 gets threaded through the knot-loop since 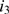 is rigidly attached to 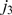, as illustrated in the rightmost panel of Figure 10. Pulling by 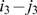 also results in generating another loop – the cysteine slip-loop – since the segment around 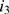 gets bent strongly to form a cigar like shape with the radius of curvature at the 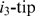 denoted by 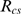. This loop extends between 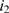 and 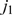. It should be pointed out that the cysteine knot-loop in the CSK is stiff whereas in a slipknotted protein (such as the thymine kinase) its size is variable (as it can be tightened on the protein backbone [40] in analogy to tightening a knot [45] by pulling).

The dynamics of pulling depends of the relationship between 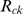 and 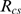 as the “cigar” may either go through or get stuck. In the former case a related force peak would arise. If the system was a homogeneous polymer, dragging would be successful when 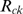 was bigger than 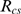. The corresponding force would be related to the work against the elasticity that was needed to bend the slip-loop to the appropriate curvature. This work is proportional to the square of the curvature. Thus the total elastic energy involved in bending the segment 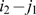 is of order 
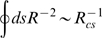
 [46], where 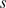 is the arc distance. Dividing this energy by the distance of pulling would yield an estimate of the force measured if thermal fluctuations were neglected. The geometrical condition for dragging in proteins is more complicated because of the presence of the side groups and the related non-homogeneities and variability across the hydrophobicity scale. The diameter of the “rope” that the knot loop is made of should not exceed the maximum a linear extension, 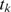 of amino acids. Thus the effective inner radius of the knot-loop is 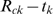. Similarly, the size of the outer circle that is tangential to the tightest slip-loop is 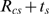, where 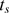 is the thickness of the slip-loop. (Both thicknesses can be considered as being site dependent and including possible hydration layer effects near polar amino acids.) Thus the slip-knot can be driven through the cystein knot-loop provided
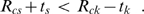
(1)In our simulations, the successful threading situations correspond to 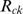 and 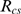 of around 7 and 3 Å. The amino acids in the knot-loop are mostly Gly, Ala, or Cys with their side groups pointing outside of the loop. One may then estimate 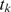 to be about 1.5 Å. On the other hand, the linear size of the amino acids in the slip-loop can be determined to be close to 2.5 Å. These estimates indicate that 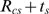 can be very close to 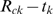 so the possibility of slipping through the knot-loop is borderline. In fact, slipping might be forbidden within the framework of the tube-picture of proteins [47],[48] in which the effective thickness of the tube is considered to be 2.7 Å.

The CSK motifs give rise to a force peak in 1vpf, 2h64(22,S), 1rv6(25,S), 1waq(26,S), 1reu(27,S), 1tgj(28), 2h62(30,S), 1tgk(31), 2c7w(38,D), 2gyr(39,S), 1lx5(95,D), and many other proteins. In these cases, the typical value of 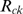 is about 7 Å. However, specificity may result in somewhat smaller values of 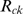 which may cause only smaller segments of the slip-loop to be threaded. If the passage is blocked, there will be no isolated force peak as happens in 1tgj and 1vpp.

#### Types of the force–displacement patterns for proteins with the disulphide bonds

In the case of proteins with very shallow cystein knot, loop or slipknot motifs, 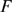 increases very rapidly with 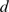 and isolated force peak does not arise (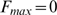). Such cases are represented, e.g., by 1bmp, 1rnr, 1ld5, and 1wzn where the slipknots are either very tight or the cystein loop is very shallow. In the case of a shallow motif, however, a force peak can sometimes be isolated as in the case of the 13th-ranked protein 1vpf (Figure 8) and in several other proteins, like 1xzg and 1dzk. In this case, the value of 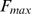 takes into account tension on the cystein bonds and it is not obvious whether such a strong elastic background should be subtracted from the value of 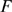 when determining 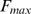 or not. In this survey, we do not subtract the backgrounds. It should be noted that in our previous surveys we missed the CSK-related force peaks because we attributed the rapid force rises at the end of pulling just to stretching of the backbone without realizing existence of structure in some such rises.

For a deep motif, the 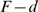 pattern may have several small force peaks before the final rise of the force, as observed for 2g4s and 1bj7. When the CSK motif is very deep, it usually does not have any influence on the shape of the 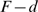 pattern apart from a much steeper final rising force. Such a situation is seen in the case of, e.g., 1j8r and 1j8s.

### Concluding remarks

This surveys identifies a host of proteins that are likely to be sturdy mechanically. Many of them involve disulphide bridges which bring about entanglements that are complicated topologically such as CSKs and CKs. The distinction between the two is that the former can depart from its native conformation and the latter cannot.

Our survey made use of a coarse grained model so it would be interesting to reinvestigate some of the proteins identified here by all-atom simulations, especially in situations when the CSK is involved. The CSK motifs may reveal different mechanical properties when studied in a more realistic model. Of course, a decisive judgment should be provided by experiment.

The very high mechanical resistance of the CSK proteins should help one to understand their biological function. The superfamily of cysteine-knot cytokines (in class small proteins and fold cystein-knot cytokines) includes families of the transforming growth-factor 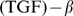 and the polypeptide vascular endothelial growth factors (VEGFs) [49],[50]. The various members of this superfamily, listed in Table 5, have distinct biological functions. For instance, VEGF-B proteins which regulate the blood vessel and limphatic angiogenesis bind only to one receptor of tyrosine kinase VEGFR-1. On the other hand, VEGF-A proteins bind to two receptors VEGFR-1 and VEGFR-2. All of these proteins form a dimer structure. The members of this familly are endowed with remarkably similar monomer structures but differ in their mode of dimerisation and thus in their propensity to bind ligands. Additionally, all dimers posses almost the same a cyclic arrangement of cysteine residues which are involved in both intra- and inter-chain disulphide bonds. These inter-chain disulphide bonds create the knot and slip-loops, where the intra-chain disulphide bonds give rise to a CSK motif when the slip-loop is gets dragged acrros the knot-loop upon pulling.

**Table 5:** Members of the cysteine-knot cytokines superfamilly.

family	domain/complex	PDB
VEGF		
	VEGF-A	1vpf*,2vpf*,1cz8,1bj1,1flt,1qty,1fpt, 1mjv,1mkg,1mkk
	VEGF-B	2c7w
	VEGF-F	1wq9,1wq8,1rv6,1fzv
TGF		
	BMP7/ActRII	1lx5,1lxi, 1m4u, 1bmp
	BMP2/IA	1reu, 1rew, 2es7, 3bmp*
	BMP2 ternary ligand-receptor complex	2h62, 2h64
	human arthemine/GFRbeta3	1tgj, 1tgk
	human arthemine/GFRalpha3	2gh0, 2gyz
human and differential factor 5		1waq , 2bhk

VEGF stands for vascular endothelial growth factor, BMP for bone morphogenetic protein, and TGF for transforming growth factor. The star 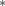 indicates uncomplexed proteins.

It has been shown experimentally [51] that such cysteine related connectivities bring the key residues involved in receptor recognition into close proximity of each other. They also provide a primary source of stability of the monomers due to the lack of other hydrogen bonds between two beta strands at the dimer interface.

The non trvial topologial connection between the monomers allow for mechanical separation of two monomers by a distance of about half of the size of the slip-loop. Our results suggest, however, that the force needed for the separation may be too high to arise in the cell.

## Materials and Methods

The input to the dynamical modeling is provided by a PDB-based structures. The structure files may often contain several chains. In this case, we consider only the first chain that is present in the PDB file. Likewise, the first NMR determined structure is considered. If a protein consists of several domains, we consider only the first of them.

The modeling cannot be accomplished if a structure has regions or strings of residues which are not sufficiently resolved experimentally. Essentially all structure-disjoint proteins have been excluded for our studies. Exceptions were made for the experimentally studied scaffoldin 1aoh and for proteins in which small defects in the established structure (such as missing side groups) were confined within cystein loops and were thus irrelevant dynamically. In these situations, the missing contacts have been added by a distance based criterion [23] in which the treshold was set at 7.5 Å. Among the test used to weed out inadequate structures involved determining distances between the consecutive 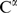 atoms. A structure was rejected if these distances were found to be outside of the range of 3.6–3.95 Å. The exception was made for prolines, which in its native state can accommodate the cis conformation. In that case, the distance between a proline 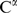 and its subsequent amino acid usually falls in the range between 2.8 and 3.85 Å. For a small group of proteins which slipped through our structure quality checking procedure, but were found to be easily fixed (e.g. 1f5f, 1fy8, and 2f3c), we used publicly avialable software BBQ [52] to rebuild locations of the missing residues. A limited accuracy of this prediction procedure seems to be adequate for our model due to its the coarse-grained nature.

The modeling of dynamics follows our previous implementations [11],[12],[18] within model 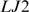 except that the contact map is as in ref. [19], i.e. with the 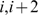 contacts excluded. There is also a difference in description of the disulphide bonds. In refs. [14],[19] they were treated as an order-of-magnitude enhancement of the Lennard-Jones contacts in all proteins. In ref. [18] the different treatment of the disulphide bonds was applied to the proteins that were found to be strong mechanically without any enhancements. Here, on the other hand, we consider such bonds as harmonic in all proteins, in analogy to the backbone links between the consecutive 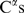. The native contacts are described by the Lennard-Jones potential 
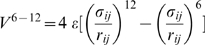
, where 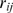 is the distance between the 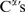 in amino acids 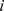 and 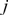 whereas 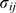 is determined pair-by-pair so that the minimum in the potential is located at the experimentally established native distance. The non-native contacts are repulsive below 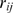 of 4 Å.

The implicit solvent is described by the Langevin noise and damping terms. The amplitude of the noise is controlled by the temperature, 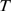. All simulations were done at 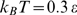, where 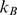 is the Boltzmann constant. Newton's equations of motion are solved by the fifth order predictor-corrector algorithm. The model is considered in the overdamped limit so that the characteristic time scale, 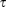, is of order 1 ns as argued in refs. [6],[53]. Stretching is implemented by attaching an elastic spring to two amino acids. The spring constant used has a value of 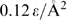 which is close to the elasticity of experimental cantilevers. One of the springs is anchored and the other spring is moving with a constant speed, 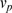. Choices in the value of the spring constant have been found to affect the look of the force-displacements patterns and thus the location of the transition state [54],[55], but not the values of 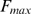 [10],[12],[18].

**Figure 11:**
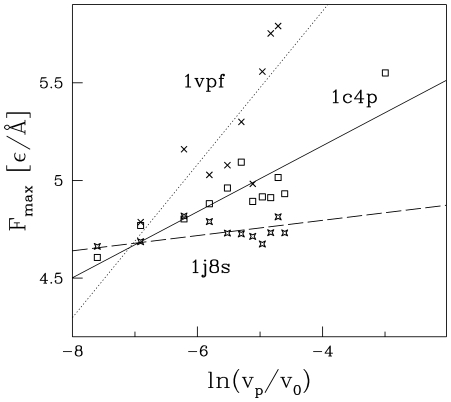
Dependence of 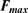 and the pulling velocity for the proteins indicated. 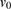
 corresponds to 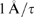 which is of order 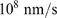. The data for several top strength proteins are shown.

The dependence on 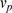 is protein-dependent and it is approximately logarithmic in 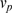 as evidenced by Figure 11 for several strong proteins. The logarithmic dependence has been demonstrated experimentally, for instance, for polyubiquitin [56],[57]. 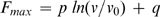. The approximate validity of this relationship is demonstrated in Figure 11 for three proteins with big values of 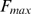. We observe that the larger the value of 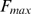, the bigger probability that the dependence on 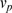 is large. When we make a fit to 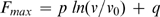 for 1vpf, 1c4p, and 1j8s, we get the parameter 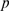 to be equal to 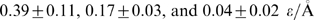 respectively (the values of 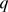 are 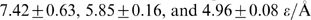 correspondingly). However, some strong proteins may have 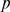 to be as low as 0.04.

When making the survey, we have used 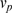 of 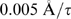 and stretching was accomplished by attaching the springs to the terminal amino acids (there is an astronomical number of other choices of the attachment points).

In order to estimate an effective experimental value of the energy parameter 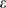, we have correlated the theoretical values of 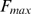 with those obtained experimentally. The experimental data points used in ref. [14] have been augmented by entries pertaining to 1emb (117–182), 1emb (182–212) [58] (where the numbers in brackets indicate the amino acids that are pulled) and 1aoh, 1g1k, and 1amu [23]. The full list of the experimental entries is provided by Table 6. Unlike the previous plots [14] that cross correlate the experimental and theoretical values of 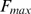, we now extrapolate the theoretical forces to the values that should be measured at the pulling speeds that are used experimentally. We assume that the unit of speed, 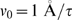, is of order 1 Å/ns and consider 10 speeds to make a fit to the logarithmic relationship. The values of parameters 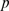 and 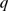 for the proteins studied experimentally are listed in Table 6.

**Table 6:** The experimental and theoretical data on stretching of proteins.

n	PDB	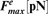	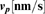	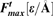	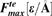	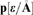	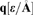	Note	Ref.
1	1tit	204+/−30	600	2.15	1.85	0.040	2.335	I27*8	[21],[22]
2	1nct	210+/−10	500	2.4+/−0.2	1.48	0.100	2.703	I54–I59	[59],[60]
3	1g1c	127+/−10	600	2.3+/−0.2	2.23	0.038	2.680	I5 titin	[61]
4	1b6i	64+/−30	1000	1.2	0.74	0.084	1.710	T4 lysozyme(21–141)	[62]
5	1aj3	68+/−20	3000	1.23	0.71	0.107	1.830	spectrin R16	[63]
6	1dqv	60+/−15	600	1.5	0.58	0.147	2.349	calcium binding C2A	[64]
7	1rsy	60+/−15	600	1.7+/−0.2	1.48	0.040	1.962	calcium binding C2A	[64]
8	1byn	60+/−15	600	1.4	1.18	0.066	1.981	calcium binding C2A	[64]
9	1cfc	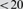	600	0.55	0.37	0.052	0.997	calmodulin	[64]
10	1bni	70+/−15	300	1.4, 1.7	1.06	0.044	1.606	barnase/i27	[65]
11	1bnr	70+/−15	300	1.05	0.71	0.053	0.053	barnase/i27	[65]
12	1bny	70+/−15	300	1.1, 1.3	0.65	0.046	0.046	barnase/i27	[65]
13	1hz6	152+/−10	700	3.5	2.79	0.064	3.542	protein L	[66]
14	1hz5	152+/−10	700	2.8	2.22	0.104	0.104	protein L	[66]
15	2ptl	152+/−10	700	2.2+/−0.2	1.88	0.045	0.045	protein L	[66]
16	1ubq	230+/−34	1000	2.32	1.47	0.134	3.019	ubiquitin	[57]
17	1ubq	85+/−20	300	0.9	0.72	0.083	1.779	ubiquitin(K48-C)*(2–7)	[56],[57]
18	1emb	350+/−30	3600	5.15+/−0.4	4.16	0.121	5.403	GFP(3–132)	[67]
19	1emb	407+/−45	12000	5.15+/−0.4	4.30	0.121	5.403	GFP(3–132)	[68]
20	1emb	346+/−46	2000	5.15+/−0.4	4.09	0.121	5.403	GFP(3–132)	[68]
21	1emb	117+/−19	3600	2.3, 4.3	1.91	0.050	2.427	GFP(3–212)	[68]
22	1emb	127+/−23	3600	2.2+/−0.2	1.51	0.164	3.197	GFP(132–212)	[68]
23	1emb	548+/−57	3600	3.5+/−0.1	2.89	0.142	4.347	GFP(117–182)	[58]
24	1emb	356+/−61	3600	3.2+/−0.2	2.94	0.075	3.709	GFP(182–212)	[58]
25	1emb	104+/−40	3600	2.3+/−0.2	1.26	0.236	3.683	GFP(N-C)	[67]
26	1fnf	75+/−20	3000	1.6, 1.8	1.70	0.130	3.069	Fniii-10	[69],[70]
27	1ttf	75+/−20	600	0.7, 1.2	0.99	0.006	1.071	Fniii-10	[71]
28	1ttg	75+/−20	600	0.7, 1.0	0.17	0.099	1.365	Fniii-10	[71]
29	1fnh	124+/−18	600	1.8	1.10	0.127	2.635	Fniii-12	[70]
30	1fnh	89+/−18	600	1.4, 1.7	1.10	0.127	2.635	Fniii-13	[70]
31	1oww	220+/−31	600	2.1+/−0.2	2.01	0.024	2.300	FNiii-1	[70]
32	1ten	135+/−40	500	1.7	1.53	0.026	1.857	TNFNiii-3	[70],[72]
33	1pga	190+/−20	400	2.4, +/−0.2	2.50	0.001	2.761	protein G	[73]
34	1gb1	190+/−20	400	1.65+/−0.2	1.69	0.045	2.237	protein G	[73]
35	1aoh	480+/−14	400	4.3+/−0.2	3.69	0.119	0.119	scaffoldin c7A	[23]
36	1g1k	425+/−9	400	3.9+/−0.01	3.22	0.028	4.106	scaffoldin c1C	[23]
37	1anu	214+/−8	400	3.3+/−0.03	2.55	0.060	3.224	scaffoldin c2A	[23]
38	1qjo	15+/−10	600	1.2	1.25	0.029	1.601	eE2lip3(N-C)	[26]

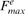
 denotes the experimentally measured value of 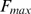 as reported in the reference stated in the last column. 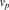 denotes the experimental pulling speed used. 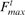 is the value of the maximal force obtained in our simulation within the 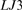 model. They were performed at 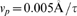. 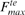 corresponds to the theoretical estimate of 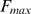 when extrapolated to the experimental speeds. The extrapolation assumes the approximate logarithmic dependence 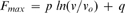, where 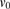 is 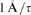. 10 speeds were used to determine the values of 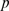 and 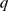 in analogy to the procedure illustrated in Figure 11 The values of 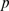 and 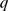 are provided in columns 7 and 8 of the Table respectively. The first column indicates the corresponding symbol that is used in Figure 12.

The main panel of Figure 12 demonstrates the relationship between the extrapolated theoretical and experimental values of 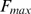. The best slope, indicated by the solid line, corresponds to the slope of 0.0091. The inverse of this slope yields 110 pN as an effective equivalent of the theoretical force unit of 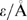. The Pearson correlation coefficient, 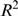 is 0.832, the rms percent error, 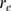, is 1.02, and the Theil 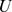 coefficient (discussed in ref. [14]) is 0.281. The inset show a similar plot obtained when the extrapolation to the experimental speeds is not done. The resulting unit of the force would be equivalent to 110 pN which differs form the previous estimate of 71 pN (shown by the dotted line in the main panel) because of the inclusion of the newly measured proteins and implementation of the extrapolation procedure. The statistical measures of error here are 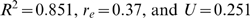. These measures are better compared to the case with the extrapolation because the extrapolation procedure itself brings in additional uncertainties. Nevertheless, implementing the procedure seems sounder physically. The spread between these various effective units of the force suggests an error bar of order 30 pN on the currently best value of 110 pN.

**Figure 12:**
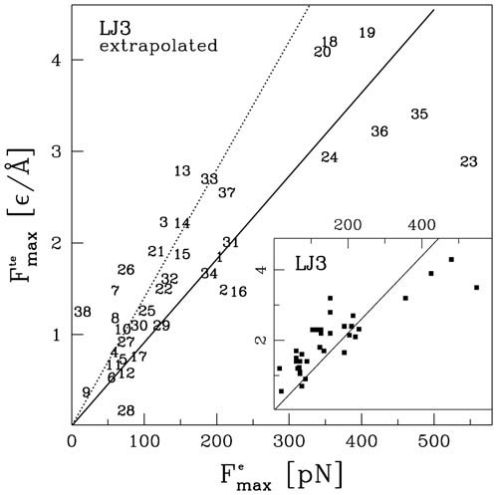
Theoretical 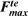 extrapolated to the pulling speeds used experimentally vs. the corresponding experimental value, 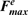. The solid line indicates the best slope of 1/(110 pN). The dotted line corresponds to the previous result of 1/(71 pN) obtained in ref. [14] where no exptrapolation was made. The inset shows a similar plot in which the extrapolation is not implemented (denoted as 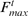 in Table 6). The list of the proteins used is provided by Table 6. It comprises almost all cases considered in ref. [14] but it also includes the recent data points obtained for the scaffoldin proteins [23] and the GFP [58]. The numerical symbols used in the Figure match the listing number in Table 6.

## Supporting Information

Figure S1 (a) Structure of trypsin 1bra (*N = 245*). The mechanically crucial disulphide bond between sites 128 and 232 is highlighted in red. (b) Structure of elastase 1elc (*N = 255*) which belongs to the same fold b.47.1.2 as 1bra. This structure does not contain two disulphide bonds that 1bra does. (c) The force-displacement plot for 1bra. *F_max_* corresponds to 3.7 *ε*/Å. The thinner line is obtained when the 128–232 disulphide bond is eliminated −*F_max_* drops to 2.7 *ε*/Å. When one more disulphide bond is cut, stretching continues to distances shown in panel (d) without affecting *F_max_*. (d) The force-displacement plot for 1elc. The corresponding *F_max_* is 2.0 *ε*/Å. In the case of 1elc, stretching results in the terminal helix pulling β strands from the inside of the protein and thus causing the inner β-barrel to unfold. If the case of 1bra (with the disulphide bridge), the terminal helix pulls the neighbouring loop. After this event, resistance grows linearly and forms one major force peak. After the peak, the whole structure opens suddenly, rupturing contacts between strands in the β-barrel and in the neighbouring loops.
(4.07 MB EPS)

Table S1 Continuation of Table 1 of the main text.
(0.04 MB PDF)

Table S2 Identification of a mechanical clamp *F_max_* for selected proteins.
(0.02 MB PDF)
