# Supplementary material for: Mechanical Strength of 17 134 Model Proteins and Cysteine Slipknots
Source: PLoS Comput Biol. 2009 Oct 30;5(10):e1000547. doi: 10.1371/journal.pcbi.1000547 (PMC2759523; doi:10.1371/journal.pcbi.1000547)
Supplement: Table S1 — Continuation of Table 1 of the main text. (0.04 MB PDF) [file pcbi.1000547.s002.pdf]

TABLE 1S: The predicted list of the strongest proteins, ctd.

n	PDBid	N	F$_{max}$ [ϵ/Å]	L_{max} [Å]	λ	CATH	SCOP
82	**2duk**	138	**3.8**	242.2	0.43		
83	**1hqp**	149	**3.8**	197.7	0.32	2.40.128.20	b.60.1.1
84	**1cuu**	197	**3.8**	421.4	0.55	3.40.50.1820	c.69.1.30
85	**1afk**	124	**3.8**	175.4	0.33	3.10.130.10	d.5.1.1
86	**2o5w**	147	**3.8**	214.8	0.33		
87	**1xzc**	197	**3.8**	422.9	0.55	3.40.50.1820	c.69.1.30
88	**1qoz**	206	**3.8**	238.7	0.29	3.40.50.1820	c.69.1.30
89	**3pcf**	200	**3.8**	251.2	0.31	2.60.130.10	b.3.6.1
90	**1odi**	234	**3.8**	458.8	0.51	3.40.50.1580	c.56.2.1
91	**1y2x**	142	**3.8**	38.1	0.01	2.60.270.20	b.97.1.2
92	**1mbq**	220	**3.8**	106.8	0.09	2.40.10.10	b.47.1.2
93	**1bj7**	150	**3.8**	195.1	0.31	2.40.128.20	b.60.1.1
94	**1odl**	234	**3.8**	458.8	0.51	3.40.50.1580	c.56.2.1
95	**1lx5**	104	**3.8**	21.7	0.01	2.10.90.10	g.7.1.3
96	**1cuz**	196	**3.8**	416.5	0.54	3.40.50.1820	c.69.1.30
97	**3pch**	200	**3.8**	252.3	0.31	2.60.130.10	b.3.6.1
98	**1oxm**	196	**3.8**	418.8	0.55	3.40.50.1820	c.69.1.30
99	**1h2p**	125	**3.8**	129.7	0.14	2.10.70.10	g.18.1.1
100	**1gwy**	175	**3.8**	143.2	0.18	2.60.270.20	b.97.1.1
101	**1cud**	197	**3.8**	420.1	0.55	3.40.50.1820	c.69.1.30
102	**1vvd**	118	**3.8**	112.1	0.14	2.10.70.10	g.18.1.1
103	**1hfh**	120	**3.8**	107.3	0.15	2.10.70.10	g.18.1.1
104	**1vvc**	118	**3.8**	112.9	0.14	2.10.70.10	g.18.1.1
105	**1cuv**	197	**3.8**	420.3	0.55	3.40.50.1820	c.69.1.30
106	**1c77**	130	**3.8**	109.5	0.18	3.10.20.130	d.15.5.1
107	**1xuk**	223	**3.8**	115.0	0.10	2.40.10.10	b.47.1.2
108	**1c2k**	223	**3.8**	114.4	0.10	2.40.10.10	b.47.1.2
109	**2stb**	222	**3.8**	113.1	0.09	2.40.10.10	b.47.1.2
110	**3tgi**	223	**3.8**	110.5	0.10	2.40.10.10	b.47.1.2
111	**3byr**	88	**3.8**	196.8	0.57		
112	**1a0j**	223	**3.8**	111.6	0.10	2.40.10.10	b.47.1.2
113	**2pcd**	200	**3.7**	251.3	0.31	2.60.130.10	b.3.6.1
114	**1vve**	118	**3.7**	104.3	0.11	2.10.70.10	g.18.1.1
115	**2pf6**	231	**3.7**	488.0	0.51		
116	**3pcl**	200	**3.7**	252.5	0.31	2.60.130.10	b.3.6.1
117	**1afl**	124	**3.7**	175.3	0.33	3.10.130.10	d.5.1.1
118	**1bs9**	207	**3.7**	241.4	0.29	3.40.50.1820	c.69.1.30
119	**1tpa**	223	**3.7**	113.4	0.10	2.40.10.10	b.47.1.2
120	**3rn3**	124	**3.7**	168.5	0.31	3.10.130.10	d.5.1.1
121	**2grk**	228	**3.7**	135.6	0.12	2.60.240.10	

122	**1xzh**	197	**3.7**	421.1	0.55	3.40.50.1820	c.69.1.30
123	**1xui**	223	**3.7**	113.7	0.10	2.40.10.10	b.47.1.2
124	**1rpg**	124	**3.7**	204.4	0.40	3.10.130.10	d.5.1.1
125	**1xuj**	223	**3.7**	115.3	0.10	2.40.10.10	b.47.1.2
126	**1bra**	223	**3.7**	112.1	0.10	2.40.10.10	b.47.1.2
127	**1rtb**	124	**3.7**	202.9	0.39	3.10.130.10	d.5.1.1
128	**1c1o**	223	**3.7**	113.8	0.10	2.40.10.10	b.47.1.2
129	**1gkg**	136	**3.7**	142.9	0.21	2.10.70.10	g.18.1.1
130	**1c5v**	223	**3.7**	115.0	0.10	2.40.10.10	b.47.1.2
131	**1tnk**	223	**3.7**	113.5	0.10	2.40.10.10	b.47.1.2
132	**1tzh**	94	**3.7**	67.4	0.10	2.10.90.10	b.1.1.1
133	**1ckl**	126	**3.7**	127.3	0.16	2.10.70.10	g.18.1.1
134	**2fwu**	157	**3.7**	88.2	0.10		b.1.27.1
135	**1aqp**	124	**3.7**	204.7	0.40	3.10.130.10	d.5.1.1
136	**2g4x**	124	**3.7**	205.1	0.40	3.10.130.10	d.5.1.1
137	**2sta**	222	**3.7**	114.3	0.10	2.40.10.10	b.47.1.2
138	**1h03**	125	**3.7**	128.9	0.14	2.10.70.10	g.18.1.1
139	**1mtv**	223	**3.7**	112.2	0.10	2.40.10.10	b.47.1.2
140	**1co7**	223	**3.7**	110.7	0.10	2.40.10.10	b.47.1.2
141	**2o1c**	147	**3.7**	215.3	0.33		
142	**1anc**	223	**3.7**	110.7	0.10	2.40.10.10	b.47.1.2
143	**1utl**	222	**3.7**	113.1	0.10	2.40.10.10	b.47.1.2
144	**1btp**	223	**3.7**	113.2	0.10	2.40.10.10	b.47.1.2
145	**1xuh**	223	**3.7**	115.4	0.10	2.40.10.10	b.47.1.2
146	**1o72**	175	**3.7**	142.9	0.18	2.60.270.20	b.97.1.1
147	**2ofc**	141	**3.7**	37.7	0.01	2.60.270.20	
148	**6rsa**	124	**3.7**	203.7	0.39	3.10.130.10	d.5.1.1
149	**2ofe**	141	**3.7**	37.8	0.01	2.60.270.20	
150	**2ofd**	141	**3.7**	37.9	0.01	2.60.270.20	
151	**2dsb**	206	**3.7**	470.0	0.58		
152	**1y3y**	223	**3.7**	111.9	0.10	2.40.10.10	b.47.1.2
153	**1xi0**	143	**3.7**	41.1	0.01	2.60.270.20	
154	**1h9i**	223	**3.7**	110.4	0.09	2.40.10.10	b.47.1.2
155	**2dsc**	195	**3.7**	429.0	0.56		
156	**1w4o**	124	**3.7**	203.3	0.39	3.10.130.10	d.5.1.1
157	**1lqe**	223	**3.7**	114.8	0.10	2.40.10.10	b.47.1.2
158	**1tgn**	222	**3.7**	112.6	0.09	2.40.10.10	b.47.1.2
159	**1tnl**	223	**3.7**	114.3	0.10	2.40.10.10	b.47.1.2
160	**1otx**	236	**3.6**	463.5	0.50	3.40.50.1580	c.56.2.1
161	**1ffe**	197	**3.6**	420.9	0.55	3.40.50.1820	c.69.1.30
162	**1bju**	223	**3.6**	113.8	0.10	2.40.10.10	b.47.1.2
163	**1anb**	223	**3.6**	111.5	0.10	2.40.10.10	b.47.1.2
164	**1ssa**	113	**3.6**	163.2	0.36	3.10.130.10	d.5.1.1

165	**1c9p**	222	**3.6**	114.1	0.10	2.40.10.10	b.47.1.2
166	**1tx6**	223	**3.6**	111.7	0.10	2.40.10.10	b.47.1.2
167	**2fws**	139	**3.6**	91.9	0.12		b.1.27.1
168	**1j16**	223	**3.6**	111.5	0.10	2.40.10.10	b.47.1.2
169	**2g4w**	124	**3.6**	204.0	0.40	3.10.130.10	d.5.1.1
170	**3pca**	200	**3.6**	251.3	0.31	2.60.130.10	b.3.6.1
171	**3pce**	200	**3.6**	251.8	0.31	2.60.130.10	b.3.6.1
172	**1fy8**	215	**3.6**	112.8	0.09	2.40.10.10	b.47.1.2
173	**3pci**	200	**3.6**	251.5	0.31	2.60.130.10	b.3.6.1
174	**1vc8**	126	**3.6**	199.1	0.37		d.113.1.1
175	**2a2g**	158	**3.6**	208.4	0.32	2.40.128.20	b.60.1.1
176	**2p78**	171	**3.6**	168.7	0.23	3.40.50.1240	
177	**1c78**	130	**3.6**	109.8	0.18	3.10.20.130	d.15.5.1
178	**1xzg**	197	**3.6**	421.8	0.55	3.40.50.1820	c.69.1.30
179	**2boc**	219	**3.6**	228.3	0.23	2.60.40.10	f.14.1.1
180	**1cuy**	197	**3.6**	420.3	0.55	3.40.50.1820	c.69.1.30
181	**2d3j**	157	**3.6**	99.8	0.07		
182	**2pqx**	245	**3.6**	147.4	0.12		
183	**1ql9**	223	**3.6**	110.7	0.10	2.40.10.10	b.47.1.2
184	**1ntp**	223	**3.6**	114.0	0.10	2.40.10.10	b.47.1.2
185	**1fmg**	223	**3.6**	115.1	0.10	2.40.10.10	b.47.1.2
186	**1sxt**	224	**3.6**	415.9	0.48	2.40.50.110	b.40.2.2
187	**1c2d**	223	**3.6**	133.6	0.12	2.40.10.10	b.47.1.2
188	**1ppe**	223	**3.6**	113.9	0.10	2.40.10.10	b.47.1.2
189	**1ane**	223	**3.6**	113.3	0.10	2.40.10.10	b.47.1.2
190	**1xzb**	197	**3.6**	421.2	0.55	3.40.50.1820	c.69.1.30

Table 1S. Continuation of Table 1 of the main text.
