# Supplementary material for: Mechanical Strength of 17 134 Model Proteins and Cysteine Slipknots
Source: PLoS Comput Biol. 2009 Oct 30;5(10):e1000547. doi: 10.1371/journal.pcbi.1000547 (PMC2759523; doi:10.1371/journal.pcbi.1000547)
Supplement: Table S2 — Identification of a mechanical clamp Fmax for selected proteins. (0.02 MB PDF) [file pcbi.1000547.s003.pdf]

TABLE 2S: Identification of a mechanical clamp F_{max} for selected proteins. F_{max} denotes the mechanical resistance obtained when all native contacts are present. F'_{max} is the force obtained when some of some sets of the relevant native contacts is removed.

rank	PDB	F_{max} [ϵ/Å]	F'_{max} [ϵ/Å]	F'_{max} [ϵ/Å]
1	1vpf	5.31	4.72 - slipknot loop	1.96 - polymer
7	2h64	4.62	4.65 - slipknot loop	2.84 - polymer
19	2c7w	4.23	4.25 - slipknot loop	2.15 - polymer
